# RNase III Domain of KREPB9 and KREPB10 Association with Editosomes in Trypanosoma brucei

**DOI:** 10.1128/mSphereDirect.00585-17

**Published:** 2018-01-17

**Authors:** Jason Carnes, Suzanne M. McDermott, Kenneth Stuart

**Affiliations:** a Center for Infectious Disease Research, Seattle, Washington, USA; b Department of Global Health, University of Washington, Seattle, Washington, USA; University at Buffalo; The Ohio State University; Texas A&M University

**Keywords:** RNA editing, RNase III, Trypanosoma brucei, editosome

## Abstract

Editosomes are the multiprotein complexes that catalyze the insertion and deletion of uridines to create translatable mRNAs in the mitochondria of kinetoplastids. Recognition and cleavage of a broad diversity of RNA substrates *in vivo* require three functionally distinct RNase III-type endonucleases, as well as five additional editosome proteins that contain noncatalytic RNase III domains. RNase III domains have recently been identified in the editosome accessory proteins KREPB9 and KREPB10, suggesting a role related to editing endonuclease function. In this report, we definitively show that KREPB9 and KREPB10 are not essential in either bloodstream-form parasites (BF) or procyclic-form parasites (PF) by creating null or conditional null cell lines. While preedited and edited transcripts are largely unaffected by the loss of KREPB9 in both PF and BF, loss of KREPB10 produces distinct responses in BF and PF. BF cells lacking KREPB10 also lack edited CYb, while PF cells have increased edited A6, RPS12, ND3, and COII after loss of KREPB10. We also demonstrate that mutation of the RNase III domain of either KREPB9 or KREPB10 results in decreased association with ~20S editosomes. Editosome interactions with KREPB9 and KREPB10 are therefore mediated by the noncatalytic RNase III domain, consistent with a role in endonuclease specialization in Trypanosoma brucei.

**IMPORTANCE** Trypanosoma brucei is a protozoan parasite that causes African sleeping sickness. U insertion/deletion RNA editing in T. brucei generates mature mitochondrial mRNAs. Editing is essential for survival in mammalian hosts and tsetse fly vectors and is differentially regulated during the parasite life cycle. Three multiprotein “editosomes,” typified by exclusive RNase III endonucleases that act at distinct sites, catalyze editing. Here, we show that editosome accessory proteins KREPB9 and KREPB10 are not essential for mammalian blood- or insect-form parasite survival but have specific and differential effects on edited RNA abundance in different stages. We also characterize KREPB9 and KREPB10 noncatalytic RNase III domains and show they are essential for editosome association, potentially via dimerization with RNase III domains in other editosome proteins. This work enhances the understanding of distinct editosome and accessory protein functions, and thus differential editing, during the parasite life cycle and highlights the importance of RNase III domain interactions to editosome architecture.

## Abstract (precis)

Trypanosoma brucei is a protozoan parasite that causes African sleeping sickness. U insertion/deletion RNA editing in T. brucei generates mature mitochondrial mRNAs. Editing is essential for survival in mammalian hosts and tsetse fly vectors and is differentially regulated during the parasite life cycle. Three multiprotein “editosomes,” typified by exclusive RNase III endonucleases that act at distinct sites, catalyze editing. Here, we show that editosome accessory proteins KREPB9 and KREPB10 are not essential for mammalian blood- or insect-form parasite survival but have specific and differential effects on edited RNA abundance in different stages. We also characterize KREPB9 and KREPB10 noncatalytic RNase III domains and show they are essential for editosome association, potentially via dimerization with RNase III domains in other editosome proteins. This work enhances the understanding of distinct editosome and accessory protein functions, and thus differential editing, during the parasite life cycle and highlights the importance of RNase III domain interactions to editosome architecture.

## INTRODUCTION

Expression of the mitochondrial genome in kinetoplastids requires posttranscriptional RNA editing to create translatable mRNAs by the insertion and/or deletion of uridines (Us), as specified by guide RNAs (gRNAs). Individual gRNAs typically direct editing at multiple editing sites (ESs), and many mRNAs require multiple gRNAs to become fully edited. Multiprotein complexes called editosomes (or RNA-editing core complexes [RECCs]) catalyze the endonucleolytic cleavage, U insertion or deletion, and subsequent ligation at each ES.

Three compositionally and functionally distinct editosomes have been characterized that are typified by distinct RNase III endonuclease and specific partner proteins (1–6). All three editosomes contain a common set of 12 proteins, which include both catalytic and noncatalytic components. Deletion ESs are edited by editosomes with KREN1 endonuclease, which also possess KREPB8 and KREX1 exoUase. Insertion ESs are edited by editosomes with either KREN2/KREPB7 or KREN3/KREPB6, but these editosomes have distinct ES preferences *in vivo* (7). Mutation of the conserved catalytic residues in RNase III domains in KREN1, KREN2, or KREN3 eliminate endonuclease function *in vivo* and *in vitro*, and all three endonucleases are essential in both bloodstream-form (BF) and procyclic-form (PF) parasites (2–4). Although all known RNase IIIs function as dimers (8), KREN1, KREN2, and KREN3 have a single RNase III domain and are present as single copies within each type of editosome, suggesting that they form heterodimers with other editosome proteins that contain RNase III motifs (1). KREPB4 and KREPB5 are among the 12 common editosome proteins, and both contain divergent, noncatalytic RNase III domains, which have been proposed as potential interactors with the three endonucleases (9–13). Recently, highly divergent RNase III domains were also identified in KREPB6, KREPB7, and KREPB8 (14, 15), which suggested that they might form dimers with their partner endonucleases instead of, or in addition to, KREPB4 and KREPB5. Like the editing endonucleases, all KREPB proteins have a U1-like Cys2His2 (C2H2) zinc finger domain N-terminal to the RNase III domain, suggesting that these proteins arise from gene duplications and have subsequently undergone specialization to perform distinct functions *in vivo*.

Editosome accessory factors KREPB9 and KREPB10 are proteins that associate with editosomes (16), and they have sequences similar to that of KREPB8. Both KREPB9 and KREPB10 have the characteristic U1-like zinc finger domains and recently identified divergent RNase III domains (15). The presence of divergent RNase III domains in KREPB9 and KREPB10 suggests that they might also functionally dimerize with other RNase III-containing editosome proteins. Indeed, recent cross-linking and mass spectrometry (CXMS) analyses of editosomes revealed proximities between KREPB10 and the RNase III-containing proteins KREN1, KREPB4, and KREPB5, in addition to KREL1 (15), but experimental validation of specific protein-protein interactions has not yet been reported.

Existing knowledge of KREPB9 and KREPB10 function is also limited. While RNA interference (RNAi) knockdown of either KREPB9 or KREPB10 produced no growth defect in PF, the extent of the knockdown was relatively weak, with only 29% of KREPB9 mRNA or 43% of KREPB10 mRNA eliminated in each cell line (16). This result therefore suggests that these proteins are not essential in PF, but it leaves the possibility that incomplete repression prevents observation of a growth defect. Real-time quantitative PCR (qPCR) analysis after RNAi knockdown showed small decreases in both preedited and edited mitochondrial transcripts. Tandem affinity purification (TAP) via tagged KREPB9 or KREPB10 revealed enrichment in editosome subcomplexes that have typical editosome exoUase and ligase activities and atypical cleavage products in endonuclease activity. The compositions of KREPB9 and KREPB10 editosome subcomplexes were also consistent with a preferential physical association for the KREX2-KREPA2-KREL1 heterotrimeric subcomplex, which is similar to results obtained with KREPB6, KREPB7, and KREPB8. Endogenous KREPB9 and KREPB10 are infrequently observed in editosome preparations via mass spectrometry (MS), and therefore they appear to be transiently or weakly associated with editosomes. KREPB9 and KREPB10 have been observed in complexes isolated via tagged KREPB5 and KREN1 in large-scale MS experiments (15). KREPB10 has also been detected in complexes with altered endonuclease compositions (either a chimeric endonuclease or KREN1 expressed in cells that repress KREPB8) or those that were isolated via tagged mitochondrial editosome-like complex-associated TUTase (MEAT1) (6, 16), which plays a poorly defined role in mRNA transcript stability (17). Therefore, the existing data suggest that KREPB9 and KREPB10 might be involved in mRNA stability and substrate recognition by editosome endonucleases.

Here, we report our investigation of KREPB9 and KREPB10 in both BF and PF cells, and we definitively show that they are not essential in BF or PF life cycle stages via our creation of either true or conditional null (CN) cell lines. Loss of KREPB9 produced little to no change in either preedited or edited transcripts in both PF and BF, while loss of KREPB10 coincided with the absence of edited CYb in BF but increased A6, RPS12, ND3, and COII edited RNAs in PF. The divergent RNase III domain in both KREPB9 and KREPB10 retains a conserved glycine that is structurally required for dimer formation, and we show that mutation of this glycine decreases the association with ~20S editosomes. These results therefore indicate that the noncatalytic RNase III domain in KREPB9 and KREPB10 promotes their association with editosomes in a manner consistent with RNase III dimerization, which suggests they may play a role in endonuclease specialization in T. brucei.

## RESULTS

**FIG 1:**
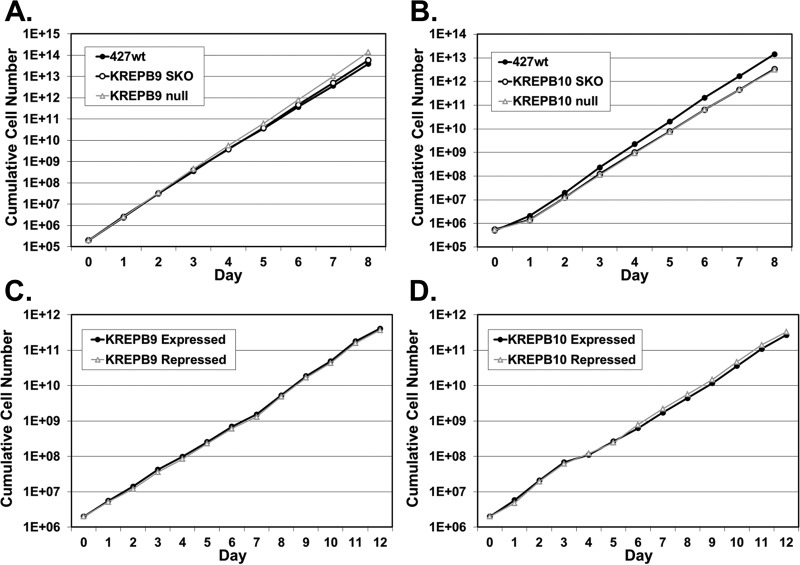
Loss of KREPB9 or KREPB10 does not alter *in vitro* growth in either BF or PF cells. (A and B) Cumulative growth of KREPB9 null BF cells (A) or KREPB10 null BF cells (B) was indistinguishable from that of cells in which a single allele was knocked out (SKO) and also similar to that in parental 427 wt cells. (D and E) Cumulative growth of PF cells in which KREPB9 (C) or KREPB10 (D) was conditionally repressed was indistinguishable from that of cells in which KREPB9 or KREPB10 was expressed.

In order to determine unambiguously if either KREPB9 or KREPB10 is essential, we used homologous recombination to generate cell lines in which expression of either gene could be eliminated. Intended homologous replacements of endogenous alleles were confirmed by PCR (data not shown). In BF, we generated KREPB9 null cells and observed negligible differences in growth compared to parental 427 wild-type (wt) cells and single-knockout cells that retained KREPB9 expression (Fig. 1A). We also generated BF KREPB10 null cells and determined that their growth was identical to single-knockout cells that retained KREPB10 expression; we observed negligible differences from 427 wt cells (Fig. 1B). The slight differences in growth for BF KREPB9 or KREPB10 null cells compared to 427 wt were of similar magnitudes as those for clonal variants with identical genetic backgrounds. In PF, we generated conditional null cell lines for either KREPB9 or KREPB10 in which a tetracycline-regulated ectopic wild-type allele was placed in the ribosomal DNA (rDNA) locus, and both endogenous alleles were eliminated. As with BF, growth of cells in which KREPB9 (Fig. 1C) or KREPB10 (Fig. 1D) was repressed was identical to that of cells in which it was expressed. Because standard PF SDM-79 growth medium contains glucose, the same growth experiments were repeated in minimal essential medium (MEM) with or without glucose to determine if oxidative phosphorylation was disrupted in the absence of either KREPB9 or KREPB10. Growth of PF cells in MEM with or without glucose again showed no differences when either KREPB9 or KREPB10 was repressed, compared to when they were expressed (data not shown). These results clearly demonstrated that neither KREPB9 nor KREPB10 is essential for *in vitro* growth of BF or PF cells.

**FIG 2:**
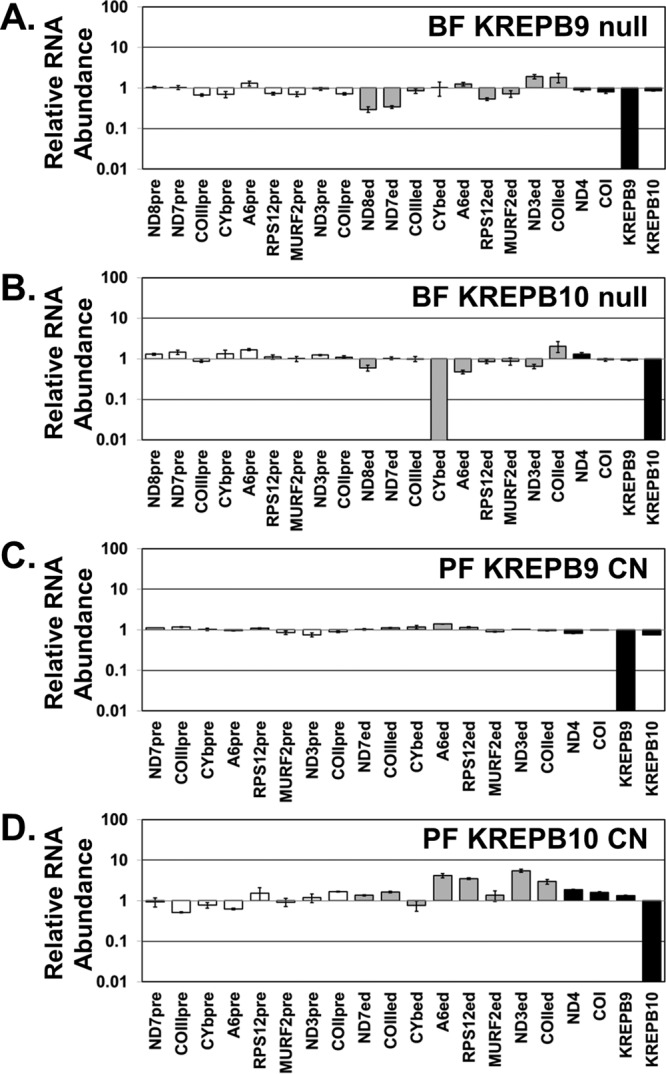
Real-time PCR analysis showed loss of KREPB9 or KREPB10 expression and their impacts on edited RNAs in both BF and PF cells. Relative RNA abundance is shown for KREPB9, KREPB10, and never-edited mRNAs COI and ND4 (black bars), preedited mRNAs (white bars), and edited mRNAs (gray bars). For each target amplicon, the relative change in RNA abundance was determined by using telomerase reverse transcriptase (tert) mRNA as an internal control, with levels in BF KREPB9 (A) and KREPB10 (B) null cell lines compared to those in 427 wt and PF CN cell lines in which either KREPB9 (C) or KREPB10 (D) was repressed, compared to levels in the same cell line in which KREPB9 or KREPB10 was expressed.

To determine the impact of KREPB9 or KREPB10 loss on edited RNAs, the steady-state abundances of several mRNA transcripts were assessed by quantitative real-time PCR. In BF, KREPB9 or KREPB10 null cells were compared to 427 wt by using telomerase reverse transcriptase (tert) mRNA as an internal reference to detect relative changes in transcript abundance. As expected, the complete loss of either KREPB9 (Fig. 2A) or KREPB10 (Fig. 2B) mRNA was observed in the respective null cell line. This result served as additional evidence that the knockout of each gene occurred as intended. Essentially no changes in the abundance of preedited, edited, or never-edited mRNAs were observed in BF KREPB9 null cells relative to abundance levels in 427 wt cells, with the exception of decreases (~70%) in edited transcripts for ND7 and ND8. Similar results were also observed in BF KREPB10 null cells, where edited CYb was undetectable and was the only transcript that was substantially altered. Expression of either wild-type KREPB9 or KREPB10 in its respective null background by introducing a constitutively expressed allele into the tubulin locus did not restore observed changes in mitochondrial mRNA abundance to the levels observed in 427 wt cells (data not shown). The reason for the decrease in edited mRNAs is therefore unclear, as these changes may not be due to loss of KREPB9 or KREPB10. Alternatively, the ectopic alleles may be inactive, or their expression levels may be insufficient. Real-time qPCR experiments were performed to analyze PF CN cell lines and compare cells in which either KREPB9 (Fig. 2C) or KREPB10 (Fig. 2D) was repressed versus cells in which they were expressed; the findings also revealed extensive loss of expression of the targeted gene. Loss of KREPB9 or KREPB10 expression after removal of tetracycline is consistent with the intended knockout of each gene and regulated ectopic expression from the rDNA locus. Loss of KREPB9 expression in PF resulted in no discernible changes to edited, preedited, or never-edited mRNAs. Loss of KREPB10 expression in PF resulted in increases of edited A6, RPS12, ND3, and COII mRNAs, while the abundance of other transcripts appeared to be unaltered. ND8 was not detected in PF by real-time PCR and is therefore omitted in Fig. 2C and D. Therefore, loss of either KREPB9 or KREPB10 or their expression in either BF or PF does not result in massive, widespread alterations to edited RNAs, which is consistent with the absence of growth defects in cells lacking either gene. The observed changes in the abundances of specific transcripts could be due to alterations in either editing or RNA stability mediated by KREPB9 or KREPB10.

**FIG 3:**
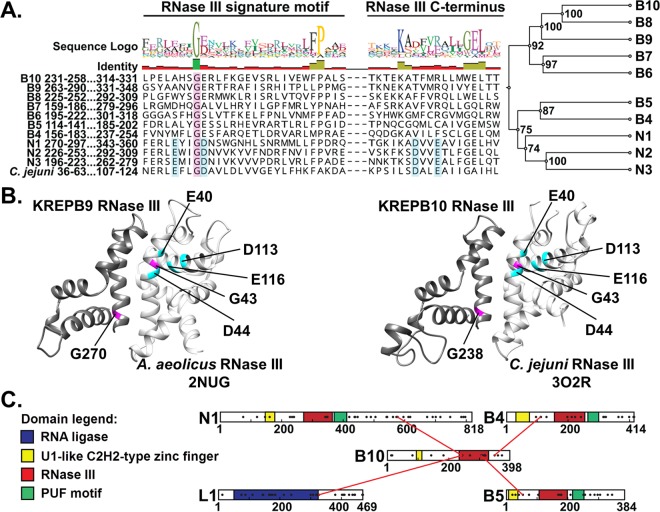
The RNase III domain identified in KREPB9 and KREPB10 conserves a structurally critical glycine residue in the signature motif. (A) Alignment of RNase III domain sequences from KREPB4- to -B10, KREN1 to -N3, and Campylobacter jejuni (GenBank accession number Q9PM40; PDB ID 3O2R). A total of 299 sequences from 35 kinetoplastid species and strains were aligned using MUSCLE. Only T. brucei sequences and the RNase III signature motif (C terminus) are shown here for clarity. Cyan shading indicates residues that aligned with those required for metal ion coordination and RNase III catalytic activity. These residues are conserved in the endonucleases KREN1 to -N3 but degenerate in KREPB4 to -B10. A universally conserved glycine is shown (shaded in magenta). An unweighted pair group method using average linkages phylogram of catalytic and noncatalytic RNase III domain-containing editosome proteins, inferred from analysis of the alignment described above, is shown. The frequencies (percentages) with which nodes were recovered in 1,000 bootstrap replications are shown. (B) Comparative modeling of KREPB9 (left) and KREPB10 (right) with the indicated bacterial RNase III crystal structures, which show critical placement of the conserved glycine residue. (C) Schematic of the location of cross-links of KREPB10 to other editosome proteins identified via CXMS. Identified cross-links fall in or near the RNase III domain.

Recent sequence analysis of KREPB9 and KREPB10 using HHpred identified highly divergent RNase III motifs in both proteins (Fig. 3A) (15). Despite the overall lack of conservation in these RNase III motifs, including the absence of residues required for catalysis, both KREPB9 and KREPB10 retain the highly conserved glycine residue homologous to G43 in archetypal bacterial RNase III. Previously published analysis of editosome interactions based on cross-linking mass spectrometry identified cross-links between KREPB10 and KREL1, KREN1, KREPB4, and KREPB5 (Fig. 3B). Critically, these cross-links occur either within or adjacent to the RNase III domain in KREPB10, and all but one of the proteins that cross-link to KREPB10 also possess an RNase III domain. These cross-links therefore suggest that this RNase III domain in KREPB10 (and perhaps the similar KREPB9) may form a structure that interacts with other editosome proteins and may form dimers with other editosome proteins that contain RNase III domains. All known RNase III proteins function as dimers, with residues in the signature motif forming an α-helix that lies parallel to the complementary α-helix as the domains pair (Fig. 3C) (8, 18). The conserved glycine residue is required for maintaining the α-helical structure within this dimerization region, due to steric limitations. This glycine is positioned on the back of the α-helix relative to the dimerization interface, packed against another α-helix in the RNase III fold, where larger amino acids cannot fit. Mutation of this glycine residue is therefore expected to disrupt the RNase III structure and interfere with the ability of the signature motif to form dimers.

To determine whether the RNase III structure in KREPB9 and KREPB10 played a role in their association with editosomes, we made cells that only express wild-type or mutant versions of these proteins in the BF null or PF CN backgrounds. The proteins were either wild type or mutated at the conserved glycine within the RNase III fold and tagged to assess their association with editosomes. We created G270R and G270V mutants of KREPB9 and G238R and G238V mutants of KREPB10 that were N-terminally V5 epitope tagged, and then we expressed them *in vivo*. Due to the steric limitations of the position of the conserved glycine, a larger, charged amino acid like arginine would be expected to create greater disruption than the smaller, uncharged valine.

**FIG 4:**
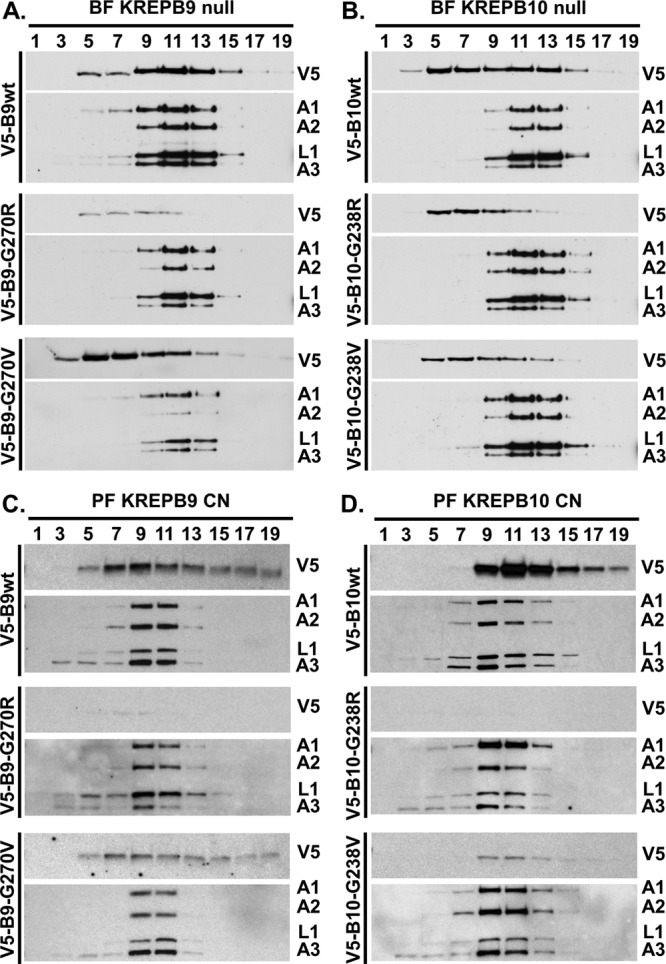
Association of KREPB9 and KREPB10 with ~20S editosomes requires the RNase III structure. Results are shown for Western analysis of glycerol gradient sedimentation using V5 antibody to detect tagged KREPB9 in BF (A) or PF (C) or tagged KREPB10 in BF (B) or PF (D). Antibodies detecting editosome proteins KREPA1, KREPA2, KREL1, and KREPA3 were used to localize the peak ~20S regions of the gradients.

In BF cells, the V5-tagged proteins were constitutively expressed from the tubulin locus in the background of the respective null cell lines. Lysates from these cells were subsequently fractionated on 10 to 30% glycerol gradients, and these fractions were analyzed by Western blotting (Fig. 4). As expected, peak signals for ~20S editosome proteins KREPA1, KREPA2, KREL1, and KREPA3 were in fractions 9 to 13 in these gradients. Probing for the V5 tag showed that the majority of wild-type KREPB9 cosedimented with ~20S editosomes in fractions 9 to 13, while the peak signal for G270R and G270V mutants sedimented in fractions 5 to 9 (Fig. 4A). Anti-V5 probing for wild-type KREPB10 revealed two peaks, one in fractions 5 to 7 and another cosedimenting in fractions 9 to 13 with ~20S editosomes. In contrast, the peak signals for G238R and G238V mutants primarily sedimented in fractions 5 to 9 (Fig. 4B). Thus, mutation to the conserved glycine within the RNase III domain in either KREPB9 or KREPB10 noticeably disrupts cosedimentation with the ~20S editosome.

In PF cells, V5-tagged KREPB9 or KREPB10 alleles were constitutively expressed from the tubulin locus in the background of the respective CN cell line in the absence of the tet-regulated expression of the wild-type allele in the rDNA locus. Western analysis of PF gradients showed the peak signal for ~20S editosome proteins KREPA1, KREPA2, KREL1, and KREPA3 in fractions 9 to 11. In contrast to BF gradients, the peak signal for wild-type KREPB9 in PF (fractions 7 to 9) only partially overlapped with ~20S editosome peak (fractions 9 to 11), and the signal for the G270V mutant did not appear to shift compared to the wild type (Fig. 4C). The Western blotting signal for the G270R mutant protein was considerably weaker due to the lower steady-state level; however, a faint signal was present in fractions 7 to 9. Thus, while mutation of KREPB9 reduced the amount of protein observed, it did not notably change gradient sedimentation. Moreover, KREPB9 appeared in many higher fractions in the gradient, including fraction 19, well beyond the typical ~20S editosome peak. Probing for KREPB10 revealed sedimentation that again appeared to only partially overlap the ~20S editosome peak (Fig. 4D). Both wild-type and G238V mutant KREPB10 had peak signal in fraction 11, while the peak ~20S editosome signal was found in fraction 9. Again, both G238V and G238R mutants had reduced steady-state levels compared to wild-type KREPB10, with the G238R mutant essentially undetectable. Similar to KREPB9, both the wild type and the G238V mutant had signal that stretched into higher sedimentation fractions, including fraction 19. Sedimentation analysis therefore revealed significant differences in KREPB9 and KREPB10 between BF and PF. Mutation of the RNase III structure disrupted cosedimentation with editosomes in BF and left protein abundance unchanged, while in PF protein abundance was reduced as the severity of the mutation increased, but sedimentation that only partially overlapped ~20S editosomes remained unchanged.

**FIG 5:**
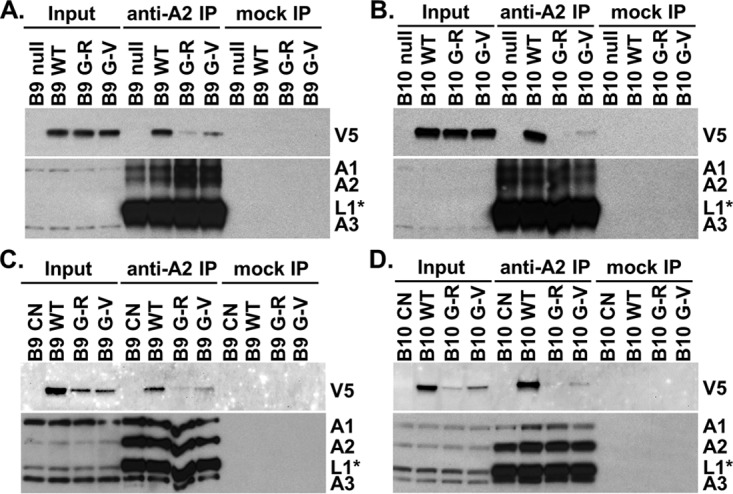
Coimmunoprecipitation analysis revealed that the intact RNase III fold is required for robust association of KREPB9 and KREPB10 with editosomes in BF and PF. Western blots were probed with anti-V5 antibody to detect V5-tagged KREPB9 or KREPB10 wt (B9 WT or B10 WT), G270R or G238R mutants (B9 or B10 G-R, respectively), or G270V or G238V mutants (B9 or B10 G-V, respectively) in both input and anti-KREPA2 immunoprecipitations (anti-A2 IP) of editosomes from BF KREPB9 (A) or KREPB10 (B) null backgrounds. Note that the V5 signal was absent in parental cells (B9 null or B10 null). Anti-KREPA1, -KREPA2, -KREL1, and -KREPA3 antibodies were used to detect editosome complexes. Similar analyses were performed in the PF CN cell line background for KREPB9 (C) or KREPB10 (D).

To more directly assess the association of KREPB9 and KREPB10 with ~20S editosomes, we isolated editosomes via anti-KREPA2 immunoprecipitation and examined the impact of mutating the conserved glycine in the RNase III domain. Editosomes isolated using anti-KREPA2 immunoprecipitation from equivalent cell numbers were analyzed by Western blotting, probing for the V5 tag on either KREPB9 or KREPB10 (Fig. 5). Simultaneous probing for KREPA1, KREPA2, KREL1, and KREPA3 served as controls for editosome immunoprecipitation and loading equivalency; note that signal from the region near KREL1 was conflated with signal from IgG heavy chain in lanes from anti-KREPA2 immunoprecipitations. Western analysis of cleared lysates from BF cell lines showed similar steady-state expression levels of mutant and wild-type KREPB9 and KREPB10 proteins, indicating roughly equivalent input into each immunoprecipitation mixture (Fig. 5A). After anti-KREPA2 immunoprecipitation of editosomes, however, the amount of V5 signal observed for the mutants was noticeably reduced, from that of the wild type, for both KREPB9 and KREPB10. In PF cleared lysates, anti-V5 Western analysis showed that the wild-type versions of KREPB9 and KREPB10 were more abundant than either mutant version, again showing that steady-state levels were reduced as a consequence of the glycine substitutions (Fig. 5B). For KREPB9 in PF, both the G270R and G270V mutant proteins appeared to be expressed at similar levels in cleared lysates, but the G270R mutant was notably reduced in amount relative to G270V in editosomes isolated by anti-KREPA2 immunoprecipitation, and both mutants were reduced compared to the wt. For KREPB10 in PF, the G238R mutant was reduced in amount relative to G238V in both cleared lysates and anti-KREPA2-isolated editosomes, and both mutants were notably reduced compared to the wt. The more pronounced reduction in signal of either KREPB9 or KREPB10 with an arginine substitution relative to the valine substitution was consistent with the predicted consequence of the mutations on the RNase III structure. These results were also consistent with the glycerol gradient results, indicating that mutation of the conserved glycine in the RNase III domain of KREPB9 or KREPB10 disrupts association with editosomes.

## DISCUSSION

We report here that neither KREPB9 nor KREPB10 is essential in BF or PF for normal growth *in vitro*. Elimination of KREPB9 or KREPB10 expression by making true null cell lines in BF or conditional null cell lines in PF resulted in limited changes in mitochondrial transcript abundance. Loss of KREPB9 coincided with no notable changes to edited RNAs in PF cells and small reductions in edited ND8 and ND7 mRNAs in BF. Because ND8 and ND7 are components of respiratory complex I, which is not essential in BF (19), this result is consistent with the lack of a growth phenotype in BF KREPB9 null cells. Loss of KREPB10 in BF coincides with a complete loss of edited CYb mRNA, which is similarly not essential in BF (20). The specific effects on ND7 and ND8 after loss of KREPB9 and on CYb after loss of KREPB10 in BF are intriguing and could reflect unknown differences among edited transcripts. However, it remains undetermined whether these reductions in specific edited transcripts are a direct consequence of the loss of KREPB9 or KREPB10. Loss of KREPB10 in PF leads to increases in edited mRNAs for A6, RPS12, ND3, and COII, a counterintuitive response that highlights the complexity of editing *in vivo*. Changes in the steady-state levels of specific mRNAs could result from KREPB9 or KREPB10 playing a role in either editing or stability of particular mRNAs. The highly divergent RNase III domain in both KREPB9 and KREPB10 retains the highly conserved glycine that is crucial for maintaining the proper positioning of the α-helices required for dimer formation. Critically, the consequences of mutating this glycine are consistent with the predicted disruption to the RNase III structure, preventing normal association of either KREPB9 or KREPB10 with ~20S editosomes. Differences between BF and PF sedimentation of KREPB9 and KREPB10 revealed resultant changes in their association with editosomes, reflecting developmental changes in how the editing machinery functions. CXMS data showed that the region in KREPB10 near and within the RNase III domain is in proximity to three other editosome proteins that contain RNase III domains, indicating a likely point of direct interaction. Because the structure of the RNase III domain in KREPB9 and KREPB10 is key to their association with editosomes, and RNase III domains are known dimerization interfaces, such interactions with other editosome proteins is strongly supported. As editosomes are faced with the formidable prospect of numerous distinct RNA substrates, KREPB9 and/or KREPB10 may function to modulate editing or stability of specific transcripts.

As previously reported, RNAi-mediated knockdown of KREPB9 or KREPB10 in PF produced no growth defect, consistent with the results reported here when we used PF CN cell lines. In contrast, different consequences to the abundance of mitochondrial mRNAs were apparent when we compared PF RNAi and CN results. While both PF RNAi and CN cells targeting KREPB9 showed little or no changes in mRNA abundance based on qPCR, results from experiments with KREPB10 PF RNAi and CN were strikingly different. After KREPB10 RNAi in PF, qPCR showed a small decrease in several mitochondrial transcripts, including never-edited, preedited, and edited mRNAs (16). qPCR data for PF KREPB10 CN cells showed never-edited and preedited mRNA levels were largely unchanged following loss of KREPB10, and there were small increases in edited mRNAs for A6, RPS12, ND3, and COII. Critically, the RNAi knockdown only partially reduced KREPB10 mRNA (by 43%), in stark contrast to its near elimination in CN cells. Therefore, subtle responses to the expression level of KREPB10 might account for some of these differences. In addition, results obtained using RNAi might be conflated with potential off-target effects that would not be seen using the CN approach. Because of these limitations to the RNAi approach, the PF results obtained with CN cells are regarded as more credible.

The observed increases in the abundances of specific edited mRNAs following loss of KREPB10 in PF are counterintuitive, as repression of most editing components results in loss of editing. One potential explanation is that KREPB10 might compete with KREPB6, -7, and -8 (KREPB6-8) *in vivo*, and the absence of this competition permits KREPB6-8 to function more efficiently, thus increasing editing. Such a competition is supported by several lines of evidence. First, KREPB8 and KREPB10 have a high degree of similarity that appears to have arisen from a gene duplication event, as they are next to each other on chromosome 8. Second, detection of KREPB10 by mass spectrometry in KREN1-TAP editosomes was promoted by KREPB8 repression (6, 16). Third, KREPB10-TAP-purified subcomplexes resembled KREPB6-8–TAP–purified subcomplexes, preferentially isolating the deletion heterotrimer of KREX2, KREPA2, and KREL1 and suggesting a similar mode of interacting with editosome proteins (16). Unlike KREPB6-8–TAP, all three editing endonucleases were detected in KREPB10-TAP-purified complexes, suggesting that KREPB10 can potentially function at both insertion and deletion ESs (16). The observed increase in edited transcripts after KREPB10 loss may, alternatively, indicate a role in PF for KREPB10 in the regulation of edited mRNA stability rather than a specific function in editing. These results are similar to those seen after knockdown of MEAT1 in PF, where multiple mitochondrial transcripts increased in abundance after MEAT1 was repressed (17). The similarity of the qPCR results, combined with the mutual presence of KREPB10 and MEAT1 in isolated editosomes (6, 16), suggests that they might have a specialized function in the regulation of RNA stability. As we observed increases in only edited mRNAs following knockdown of KREPB10, and short A-tails are known to differentially affect stability of edited and preedited messages (21, 22), it is possible that KREPB10 interacts with these short tails to modulate RNA stability.

Interestingly, KREPB9 and KREPB10 are conserved and syntenic in most trypanosomatids, but they are absent in *Leishmania* species. This is contrast to the 19 editosome complex proteins identified in T. brucei, of which all but 1 (KREPA5) are found in *Leishmania*. Although these highly diverged kinetoplastids all perform RNA editing, 1,354 canonical ESs are found in T. brucei but only 629 ESs are found in Leishmania tarentolae. Thus, the correlation of increased editing and editing site diversity with the presence of KREPB9 and KREPB10 genes within kinetoplastid species is consistent with a role in RNA processing, either via editing or stability, which in turn could influence metabolic changes between life cycle stages.

A growing body of evidence demonstrates that the RNA editing machinery differs functionally between BF and PF, and the mechanism underlying the developmental changes remains unknown. While several differences in RNA editing at the transcript level have been known for decades, only recently has it become clear that essential editosome proteins, such as KREPA3 and KREPB5, have distinct amino acid requirements in different life cycle stages (9–11). The distinct sedimentation profiles of KREPB9 and KREPB10 in BF compared to PF further indicate that the editing machinery has developmental differences and raise the possibility that KREPB9 or KREPB10 might have specialized roles in other life cycle stages, such as metacyclics or stumpy forms. As the mechanism responsible for developmental regulation of RNA editing has long remained elusive, examination of these life cycle-dependent functional differences in editosome proteins may be the key to unraveling the mystery.

The identification of divergent RNase III domains in KREPB9 and KREPB10 has provided a new perspective into their function. Previous bioinformatics analyses indicated the presence of a WGR motif in the same region where the newly identified RNase III domain resides (16). The identification of the WGR motif now appears to have been spurious, as we showed here that the RNase III domain functions in association with editosomes. This RNase III domain proved difficult to detect in part because the catalytic amino acids are not conserved, which indicates that KREPB9 and KREPB10 do not directly cleave substrate RNA. All editosome KREPB paralogs therefore contain divergent, noncatalytic RNase III domains, in contrast to the catalytic RNase III domains in the three KREN paralogs. All KREPB and KREN RNase III domains retain a highly conserved glycine that is crucial for maintaining the proper positioning of α-helices required for dimer formation. Replacement of this glycine in KREPB9 and KREPB10 prevents normal association of either KREPB9 or KREPB10 with ~20S editosomes. This is consistent with previous work, where the same conserved residue was important for KREPB4 and KREPB5 *in vivo* function and editosome association in both PF and BF (9–12), and for PF KREN1 and KREN2 function *in vitro* (5). Together these results suggest that KREPB9 and KREPB10 are components of multiple different potential RNase III heterodimers within editosomes, which is further indicated by proximity of the KREPB10 RNase III domain to the other RNase III editosome proteins, KREN1, KREPB4, and KREPB5, in CXMS (15). Different heterodimers may form at different times during cycles of editing or in different life cycle stages, or they may contribute to recognition and cleavage of the numerous similar but distinct insertion and deletion editing sites. Evidence is now mounting that the endonuclease step in the catalytic cycles of editosomes is more complex than expected, and the presence of multiple proteins containing noncatalytic RNase III domains provides a way to handle that complexity.

## MATERIALS AND METHODS

### Growth of cells *in vitro*.

BF cells were grown in HMI-9 (23) with 10% fetal bovine serum (FBS) at 37°C, 5% CO_2_. PF cells were grown at 27°C in SDM-79 (24) with 10% FBS, except for growth curve analyses, for which they were grown in MEM-Pros (25) with 10% dialyzed FBS plus or minus 6 mM d-glucose (26). For growth curve analyses, BF were reseeded at 2 × 10^5^ cells/ml in 5 ml every day and PF were reseeded at 2 × 10^6^ cells/ml in 10 ml every 2 days, and cell densities were measured using a Coulter Counter. Transfections of BF cell lines with the Amaxa Nucleofector (Lonza) and of PF cell lines with the BTX transfection device (Harvard Apparatus, Inc.) were carried out as described previously (27). Concentrations of drugs used for selection and tet-regulated expression of transgenes were as follows: BF were selected in 1 to 1.5 µg/ml hygromycin, 0.1 µg/ml puromycin, 5 µg/ml ganciclovir; PF were selected in 15 µg/ml G418, 25 µg/ml hygromycin, 2.5 µg/ml phleomycin, 0.5 µg/ml tet, 1 µg/ml puromycin, 10 µg/ml blasticidin, 25 µg/ml ganciclovir.

### Generation of transgenic cell lines.

DNA constructs for KREPB9 or KREPB10 allele knockouts in the BF 427 wt background were generated by PCR amplification of puromycin and hygromycin drug cassettes from SM07 (27) and pyrFEKO-HYG (Addgene plasmid 24020; George Cross), respectively. Targeting sequences were also amplified using sequences described in Table S1 in the supplemental material and combined with drug cassettes in fusion PCRs (27). The resulting constructs were transfected stepwise into the BF 427 wt cell line, transgenic lines were selected by puromycin and hygromycin resistance, and correct insertion of knockout cassettes was assessed by PCR. To create regulatable ectopic expression of KREPB9 or KREPB10 in PF, wild-type open reading frames containing stop codons were flanked in frame with *att*B (Gateway) recombination sites and PCR amplified using primers described in Table S1. BP Clonase II (Thermo Fisher Scientific) was used to transfer the PCR product into the Gateway entry vector pDONR221. LR Clonase II (Thermo Fisher Scientific) was then used to transfer the KREPB9 or KREPB10 sequence into the Gateway destination vector pLEW100v5(BLE)GW (27), which allows for tet-regulatable expression of wt KREPB9 or KREPB10 from the rRNA locus. The resulting pLEW100v5(BLE)-KREPB9 and pLEW100v5(BLE)-KREPB10 plasmids were linearized with NotI and transfected into PF 29.13 cells, and transgenic lines were selected by phleomycin resistance. Preparation of DNA constructs to eliminate endogenous KREPB9 or KREPB10 alleles in the regulatable PF cell lines were generated by PCR amplification of blasticidin and puromycin drug cassettes from SM06 and SM07 (27), respectively, using primers described in Table S1. Transgenic cell lines were selected by blasticidin and puromycin resistance, and correct insertion of knockout cassettes were assessed by PCR as previously described for the generation of the PF KREPB5 CN cell line (10). The drug selection cassettes in the resulting BF null and PF CN cell lines were excised using transient expression of Cre recombinase from undigested pLEW100Cre_del_tetO (Addgene plasmid 24019; George Cross), modified by removal of the phleomycin drug cassette, and selected with ganciclovir (InvivoGen) as previously described (10). For generation of exclusive expression cell lines, pENTR-KREPB9 and pENTR-KREPB10 were used in LR reactions with the destination vector pHD1344tub(PAC)GW-Nterm3V5, which allows for constitutive expression of N-terminal 3×V5-tagged KREPB9 or KREPB10 in the β*-*tubulin locus. This destination vector also places the mitochondrial targeting sequence from T. brucei dihydrolipoyl dehydrogenase (LipDH) on the N terminus of the 3×V5 tag to ensure mitochondrial localization. Primers used for the creation of this vector from the original pHD1344tub(PAC) are described in Table S1. The resulting pHD1344tub(PAC)-Nterm3V5-KREPB9 and pHD1344tub(PAC)-Nterm3V5-KREPB10 plasmids were used as the template for site-directed mutagenesis (QuikChange II kit; Agilent) using forward and reverse primers listed in Table S1. NotI-digested plasmids were transfected into the appropriate BF null and PF CN cell lines. Transgenic lines were selected by puromycin resistance, and constitutive expression of 3×V5-KREPB9 or 3×V5-KREPB10 was confirmed by Western blotting.

10.1128/mSphereDirect.00585-17.1 TABLE S1 Oligonucleotide sequences used in this study. Download TABLE S1, PDF file, 0.2 MB. Copyright © 2018 Carnes et al. 2018 Carnes et al. This content is distributed under the terms of the Creative Commons Attribution 4.0 International license.

### Fractionation on glycerol gradients.

Glycerol gradient fractionation was carried out on total cell lysates from 2 × 10^9^ PF or BF cells in the presence or absence of 0.5 µg/ml tet. Following lysis in 650 µl lysis buffer (10 mM Tris-HCl [pH 7.2], 10 mM MgCl_2_, 100 mM KCl, 1% Triton X-100) and centrifugation (16,000 × *g*, 10 min, 4°C), cleared lysates were loaded onto 11-ml 10-to-30% glycerol gradients and centrifuged at 38,000 rpm for 8 h at 4°C in an SW40Ti rotor (Beckman). A total of 24 fractions of 500 µl were collected from top to bottom, flash-frozen in liquid nitrogen, and stored at −80°C.

### Immunoprecipitation.

For each immunoprecipitation mixture, 0.5 ml of P1H3-D7 mouse monoclonal supernatant containing anti-KREPA2 antibody (28) was incubated with 40 μl goat anti-mouse IgG M450 Dynabeads (Invitrogen), incubated at 4°C for 2 h, then washed 3 times with 1 ml IPP150 (10 mM Tris-HCl [pH 8.0], 150 mM NaCl, 0.1% Nonidet P-40) (28). Cleared lysate was prepared by lysis of 2 × 10^8^ cells in 1 ml IPP150 (10 mM Tris-HCl [pH 8.0], 150 mM NaCl, 0.1% Nonidet P-40, Complete protease inhibitors [Roche]) with 1% Triton X-100, followed by centrifugation at 16,000 × *g* at 4°C. Anti-KREPA2 immunoprecipitations were carried out by incubation of 0.5 ml cleared lysate (1 × 10^8^ cells) with either anti-KREPA2 or mock (no antibody) Dynabeads. After incubation, the supernatant was removed and beads were washed four times with 1 ml IPP150. Complexes bound to beads were eluted with 100 μl of 2× SDS sample buffer that was heated for 5 min at 95°C.

### SDS-PAGE and Western blotting.

SDS-PAGE loading buffer was added to cleared whole-cell lysates or to samples containing purified protein complexes and resolved on 10% SDS-PAGE gels (Criterion Tris-HCl; Bio-Rad). For Western analysis, resolved proteins were transferred to Immobilon-P polyvinylidene difluoride membranes (Millipore) and probed using the antibodies described in Table S2. Generally, goat anti-mouse IgG–horseradish peroxidase (HRP) secondary antibodies were used, except where mouse anti-V5 primary antibodies were used to probe Western blots of anti-KREPA2-immunoprecipitated proteins. Here, protein A-conjugated HRP was employed to prevent detection of denatured anti-KREPA2 IgG heavy chain. Blots were developed with an enhanced chemiluminescence kit (Thermo Scientific) per the manufacturer’s instructions and imaged using the FluorChem E system (ProteinSimple) or X-ray film (Kodak).

10.1128/mSphereDirect.00585-17.2 TABLE S2 Antibodies used in this study. Download TABLE S2, PDF file, 0.1 MB. Copyright © 2018 Carnes et al. 2018 Carnes et al. This content is distributed under the terms of the Creative Commons Attribution 4.0 International license.

### RNA isolation and RT-qPCR analysis.

Total RNA was harvested using TRIzol and treated with Turbo DNase (Life Technologies, Inc.), following the manufacturer’s instructions. RNA integrity was confirmed using an RNA nanochip on a BioAnalyzer (Agilent Technologies). cDNA was generated from 1 to 2 µg of total RNA by using TaqMan reverse transcription reagents with MultiScribe reverse transcriptase (Life Technologies, Inc.) and subsequently preamplified in multiplex specific target amplification (STA) reactions using TaqMan PreAmp master mix (Life Technologies, Inc.). STA reactions were treated with exonuclease I (NEB) prior to qPCR. The abundance of reference, never-edited, preedited and edited transcripts were then determined using high-throughput real-time qPCR on the BioMark HD system as previously described (10). Primers are described in Table S1. Calculations of RNA levels in BF were performed to levels in null cell lines and 427 wt cells, while PF levels were compared to those in CN cell lines after tet withdrawal (repressed) relative to the presence of tet (expressed); both BF and PF analyses were performed using the 2^−ΔΔ*CT*^ method (29) with tert as an internal reference (30). Either two or four technical replicates of each cDNA sample were assayed for each target and internal reference per experiment, and threshold cycle (*C*_*T*_) data were averaged before performing the 2^−ΔΔ*CT*^ calculations. Experiments were repeated using two (PF) or three (BF) biological replicates.

### Homology modeling.

Template identification and model generation were carried out using HHPred and Modeler (31, 32). Structures and comparative models were visualized using Chimera (33, 34).
