# Supplementary material for: RNase III Domain of KREPB9 and KREPB10 Association with Editosomes in Trypanosoma brucei
Source: mSphere. 2018 Jan 17;3(1):e00585-17. doi: 10.1128/mSphereDirect.00585-17 (PMC5770545; doi:10.1128/mSphereDirect.00585-17)
Supplement: TABLE S1 [file sph001182459st1.pdf]

Table S1.

Primer Description	Primer sequence	Reference
KREPB9 forward primer with *att*B1 site	GGGGACAAGTTTGTACAAAAAAGCAGGCTCCAAAATGTCACTCAGGTCCACAAT	This study
KREPB9 reverse primer (plus stop codon) with *att*B2 site	GGGGACCACTTTGTACAAGAAAGCTGGGTCTCAGCAAAAGGTGCTTTCCTC	This study
KREPB10 forward primer with *att*B1 site	GGGGACAAGTTTGTACAAAAAAGCAGGCTCCAAAATGATGTACCGGTGGAGCC	This study
KREPB10 reverse primer (plus stop codon) with *att*B2 site	GGGGACCACTTTGTACAAGAAAGCTGGGTCTCACGCTGCGCCTATTAATACC	This study
Forward primer floxed cassette amplification from SM06, SM07, and pyrFEKO-HYG (1)	GATAAGCTTATAACTTCGTATAGCATACA	27
Reverse primer floxed cassette amplification from SM06, SM07, and pyrFEKO-HYG (1)	GACCCACTTAGGATCCATAACTTCGTA	27
KREPB9 knockout outer 5' fragment forward	AGAACACCAGGATGGAAGAG	This study
KREPB9 knockout outer 5' fragment forward nested	GGTATACTCTTCCAGAGCAC	This study
KREPB9 knockout outer 5' fragment reverse	ATACGAAGTTATAAGCTTATCAGTACATTATGACGTATGTTCGCA	This study
KREPB9 knockout inner 5' fragment forward	GTGTCTTGCCGCATTACCAA	This study
KREPB9 knockout inner 5' fragment reverse	ATACGAAGTTATAAGCTTATCGAGGGTCCTACCGCATGAAC	This study
KREPB9 knockout outer 3' fragment forward	TTATGGATCCTAAGTGGGTCTTGGGTGGTGGACGGTTTG	This study
KREPB9 knockout outer 3' fragment reverse	GATTCCTTGCGTTTTCCATCGT	This study
KREPB9 knockout outer 3' fragment reverse nested	TGAGAGGGGATGTGTTAGACAG	This study

KREPB9 knockout inner 3' fragment forward	TTATGGATCCTAAGTGGGTCCTCGGAGGAACGAGTGTAGC	This study
KREPB9 knockout inner 3' fragment reverse	CGGGTGTCACGACCTTTGAA	This study
KREPB10 knockout outer 5' fragment forward	GGCTGGCAACCGTACGAAAG	This study
KREPB10 knockout outer 5' fragment forward nested	AAACACATGGCGGATCTTGC	This study
KREPB10 knockout outer 5' fragment reverse	ATACGAAGTTATAAGCTTATCCGTGAACGTTACTGTACACAG	This study
KREPB10 knockout inner 5' fragment forward	TGTACAGTAACGTTCACGCCA	This study
KREPB10 knockout inner 5' fragment reverse	ATACGAAGTTATAAGCTTATCTCGTTATCAATCTCACACGGACA	This study
KREPB10 knockout outer 3' fragment forward	TTATGGATCCTAAGTGGGTCTTTTCTCCCATTCTGCATTCTTTT	This study
KREPB10 knockout outer 3' fragment reverse	CAACGCCACCATCAACTGTAA	This study
KREPB10 knockout outer 3' fragment reverse nested	ACCCAAGTGACTTTTCCTGCT	This study
KREPB10 knockout inner 3' fragment forward	TTATGGATCCTAAGTGGGTCTGCTCCCCGCGAAATTCTTA	This study
KREPB10 knockout inner 3' fragment reverse	AGCAATGGTGGTAGCAAAAGG	This study
KREPB9 G270V site-directed mutagenesis	CGGAAAGTGCGCTCAACAACGTTTGCTGCGT	This study
KREPB9 G270V site-directed mutagenesis	ACGCAGCAAACGTTGTTGAGCGCACTTTCCG	This study
KREPB9 G270R site-directed mutagenesis	AGTGCGCTCACGAACGTTTGCTGCGTAGCT	This study
KREPB9 G270R site-directed mutagenesis	AGCTACGCAGCAAACGTTCGTGAGCGCACT	This study
KREPB10 G238V site-directed mutagenesis	CTTGAAAAGTCGTTCGACGCTGTGCGCCAACTC	This study

KREPB10 G238V site-directed mutagenesis	GAGTTGGCGCACAGCGTCGAACGACTTTTCAAG	This study
KREPB10 G238R site-directed mutagenesis	GAAAAGTCGTTCGCGGCTGTGCGCCAACT	This study
KREPB10 G238R site-directed mutagenesis	AGTTGGCGCACAGCCGCGAACGACTTTTC	This study
TERT BioMark forward	GAGCGTGTGACTTCCGAAGG	30
TERT BioMark reverse	AGGAACTGTCACGGAGTTTGC	30
KREPB9 ORF BioMark forward	TCCGAATGCGATGCTAGAGA	16
KREPB9 ORF BioMark reverse	CGCACCTTCATTTGCATGT	16
KREPB10 ORF BioMark forward	CGCTGAGTAACTTGCGTTTGG	16
KREPB10 ORF BioMark reverse	AGGCGCTCCGGCACAT	16
COI BioMark forward	CCCGATATGGTATTTCCTCGTATAAA	3
COI BioMark reverse	CCCCCATACCCTCTTCAGTCA	3
ND4 BioMark forward	CAATCTGACCATTCCATGTGTGA	2
ND4 BioMark reverse	TTTCAGCACAATACTTGCTAATAAAACA	2
A6 BioMark pre-edited forward	TTGCCTTTGCCAAACTTTTAGAAG	2
A6 BioMark pre-edited reverse	ATTCTATAACTCCAAAATCACAACTTTCC	2
CYb BioMark pre-edited forward	ATATAAAAGCGGAGAAAAAAGAAAG	2
CYb BioMark pre-edited reverse	CCCATATATTCTATATAAACAACCTGACA	2
COII BioMark pre-edited forward	ATTACAGTGTAACCATGTATTGACATT	2
COII BioMark pre-edited reverse	TTCATTACACCTACCAGGTTCTCT	2
COIII BioMark pre-edited forward	GAAACCAGATGAGATTGTTTGCA	2
COIII BioMark pre-edited reverse	TTCATTCCAACTAAACCCTTTCC	2
MURF2 BioMark pre-edited forward	GATTTTAAGATTGGCTTTGATTGA	2

MURF2 BioMark pre-edited reverse	AATATAAAATCTAGATCAAACCATCACA	2
RPS12 BioMark pre-edited forward	CGACGGAGAGCTTCTTTTGAATA	2
RPS12 BioMark pre-edited reverse	CCCCCCACCCAAATCTTT	2
ND3 BioMark pre-edited forward	GAATGGGAGATGGGTTTTGG	2
ND3 BioMark pre-edited reverse	AACAAATCTCTTTACCCCCTTCAG	2
ND7 BioMark pre-edited forward	GCGGGCGGAGCATTATT	2
ND7 BioMark pre-edited reverse	GATCTACGGTCCCCTCTTTCCT	2
A6 BioMark edited forward	GATTTATTTTGGTTGCGTTTGTTATTATG	2
A6 BioMark edited reverse	CAAACCAACAAACAAATACAAATCAAAC	2
CYb BioMark edited forward	AAATATGTTTCGTTGTAGATTTTTATTATTT	2
CYb BioMark edited reverse	CCCATATATTCTATATAAACAACCTGACA	2
COII BioMark edited forward	ATTACAGTGTAACCATGTATTGACATT	2
COII BioMark edited reverse	ATTTCATTACACCTACCAGGTATACAA	2
COIII BioMark edited forward	TTGTGTTTTATTACGTTGTATCCAGTATTG	2
COIII BioMark edited reverse	CGAAAGCAAACTCACAACACAAA	2
MURF2 BioMark edited forward	GATTTTAATGTTTGGTTGTTTTAATTTAG	2
MURF2 BioMark edited reverse	AATATAAAATCTAGATCAAACCATCACA	2
RPS12 BioMark edited forward	CGTATGTGATTTTTGTATGGTTGTTG	2
RPS12 BioMark edited reverse	ACACGTCGGTTACCGGAACT	2
ND3 BioMark edited forward	TGTTTTCGTTGTTGTTTGTGGTT	2
ND3 BioMark edited reverse	CAATGTATAAAACACCAAACGTGAATT	2
ND7 BioMark edited forward	GCATCCCGCAGCACATG	2
ND7 BioMark edited reverse	CTGTACCACGATGCAAATAACCTATAAT	2

HindIII-LipDH mito targeting sequence- N-terminal 3xV5 cloning forward primer	GATCAAGCTTATGTTCCGTCGCTGCTTTCCGATCTTTAACCCCTACGACGTC-GGTAAGCCTATCCCTAACC	This study
3xV5-XhoI-BglII cloning reverse primer	GATCAGATCTCTCGAGCGTGCTATCAAGACCGAGGA	This study
Reading frame B for Gateway conversion of pHD1344tub(PAC)-Nterm3V5 (cloned into blunted XhoI site introduced using primers above)	ATCTCAACAAGTTTGTACAAAAAA-[CmR-ccdB]-TTTCTTGTACAAAGTGGTTGAT	ThermoFisher Scientific Gateway Vector Conversion System

References

2. Carnes J, Trotter JR, Ernst NL, Steinberg A, Stuart K. 2005. An essential RNase III insertion editing endonuclease in Trypanosoma brucei. Proc Natl Acad Sci U S A 102:16614-9.

3. Carnes J, Trotter JR, Peltan A, Fleck M, Stuart K. 2008. RNA editing in Trypanosoma brucei requires three different editosomes. Mol Cell Biol 28:122-30.

16. Lerch M, Carnes J, Acestor N, Guo X, Schnaufer A, Stuart K. 2012. Editosome accessory factors KREPB9 and KREPB10 in Trypanosoma brucei. Eukaryot Cell 11:832-43.

27. Merritt C, Stuart K. 2013. Identification of essential and non-essential protein kinases by a fusion PCR method for efficient production of transgenic Trypanosoma brucei. Mol Biochem Parasitol 190:44-9.

30. Brenndorfer M, Boshart M. 2010. Selection of reference genes for mRNA quantification in Trypanosoma brucei. Mol Biochem Parasitol 172:52-5.
