# Supplementary material for: RNase III Domain of KREPB9 and KREPB10 Association with Editosomes in Trypanosoma brucei
Source: mSphere. 2018 Jan 17;3(1):e00585-17. doi: 10.1128/mSphereDirect.00585-17 (PMC5770545; doi:10.1128/mSphereDirect.00585-17)
Supplement: TABLE S2 [file sph001182459st2.pdf]

Table S2.

Antibody name	Raised in	Dilution	Source
V5 Epitope Tag Monoclonal Antibody	Mouse	1 µg per IP with 1 × 10^8 cells<br>1/5000 WB	Life Technologies<br>R960-25
KREPA1<br>Monoclonal Antibody	Mouse	1/25 WB	28<br>P4D8-F6
KREPA2<br>Monoclonal Antibody	Mouse	1 mL per IP with 1 x 10^8 cells<br>1/12.5 WB	28<br>P1H3-D7
KREL1<br>Monoclonal Antibody	Mouse	1/50 WB	28<br>P3C1-G2
KREPA3<br>Monoclonal Antibody	Mouse	1/25 WB	28<br>P3C12-B6
Goat-Anti-Mouse-IgG-HRP	Goat	1/5000 WB	Bio-Rad
Protein A, HRP conjugate	-	1/5000 WB	Millipore-Sigma

References

28. Panigrahi AK, Gygi SP, Ernst NL, Igo RP, Jr., Palazzo SS, Schnaufer A, Weston DS, Carmean N, Salavati R, Aebersold R, Stuart KD. 2001. Association of two novel proteins, TbMP52 and TbMP48, with the Trypanosoma brucei RNA editing complex. Mol Cell Biol 21:380-9.
